# Periodontal cell mechanotransduction

**DOI:** 10.1098/rsob.180053

**Published:** 2018-09-12

**Authors:** Sasanka S. Chukkapalli, Tanmay P. Lele

**Affiliations:** 1 Department of Oral Biology, University of Florida, College of Dentistry, Gainesville, FL 32610, USA; 2 Center for Molecular Microbiology, University of Florida, College of Dentistry, Gainesville, FL 32610, USA; 3 Department of Chemical Engineering, University of Florida, Gainesville, FL 32611, USA

**Keywords:** mechanobiology, mechanotransduction, cytoskeleton, periodontal disease, cell shape, traction force

## Abstract

The periodontium is a structurally and functionally complex tissue that facilitates the anchorage of teeth in jaws. The periodontium consists of various cell types including stem cells, fibroblasts and epithelial cells. Cells of the periodontium are constantly exposed to mechanical stresses generated by biological processes such as the chewing motions of teeth, by flows generated by tongue motions and by forces generated by implants. Mechanical stresses modulate the function of cells in the periodontium, and may play a significant role in the development of periodontal disease. Here, we review the literature on the effect of mechanical forces on periodontal cells in health and disease with an emphasis on molecular and cellular mechanisms.

## 1. Introduction

The human periodontium is a structure that supports and anchors teeth to marginal gingiva and to alveolar bone. The gingiva and periodontal ligaments consist of stem cells, fibroblasts and epithelial cells [1]. These cells are exposed to mechanical stresses [2] generated by biological process such as the chewing motions of teeth [3], by flows generated by tongue motions [4] and by orthodontic forces generated by implants [5]. These mechanical forces modulate the functions of periodontal cells [6,7] through a process broadly called ‘cellular mechanotransduction’ [8]. Much recent research has focused on the molecular mechanisms of periodontal cell mechanotransduction.

The periodontium also hosts a diverse range of oral microflora [9,10], which continually initiate an inflammatory cascade in the periodontal tissue environment [11]. Some flora might become dominant, causing dysbiosis, a loss of equilibrium, ultimately causing chronic periodontal disease [12,13]. Periodontal disease (PD) caused by bacterial infection is a major cause of teeth and alveolar bone loss and is associated with systemic inflammation and cardiovascular disease [14]. Seventy per cent of adults 65 years and older have some form of PD [15]. PD is characterized by a change in tissue structure through degradation and remodelling of the extracellular matrix due to bacterial activity and gingival inflammation and degradation of bone and teeth. Bacterial infections trigger inflammatory cascades in PD, cause changes in local cytokine secretion, degrade collagen fibres, alter fibroblast cell structure and result in gingival detachment from the cementum and alveolar bone resorption [13,14,16–18].

Mechanical forces in the periodontium and the physical properties of periodontal cells together may be a complicating factor in the progression of PD. Here, we summarize effects of the different types of mechanical loading on periodontal cell function and discuss them in the context of PD.

## 2. Effect of mechanical loading on periodontal cell proliferation

Of all the periodontal cell types, periodontal ligament fibroblasts (PDLFs) have probably received the most attention in terms of how they respond to mechanical forces. PDLFs are an important component of the periodontal ligament (PDL) [19]. The PDLs are tensile cable-like structures that connect alveolar bone with cementum on teeth [20]. In healthy humans, mechanical load on teeth during chewing compresses or stretches the PDL [6]. PDLFs secrete collagen [20], and organize it into collagen fibres which form the main load-bearing structure in PDLs [21]. The collagen fibres attach on one end to alveolar bone and the other end is inserted into cementum [21], and are referred to as Sharpey's fibres [22]. PDLFs are also responsible for repair of the PDLs [23].

PDLFs generate tensile forces themselves [24], and are subject to significant mechanical stresses during expansion and relaxation of the PDL caused by normal motions of the jaw during chewing. Multiple approaches have been used to apply forces to periodontal cells *in vitro* in order to explore how these cells might respond to physiological forces [25]. A widely used approach has involved culturing these cells on flexible membranes coated with appropriate matrix proteins (such as collagen or fibronectin), and applying controlled strain to the membranes. The strain magnitude and strain frequency are parameters that are set typically with computer-based control. Strains can be applied from a few hours to several days. Furthermore, depending on the geometry of the underlying membrane and the force applied, the strains can be uniaxial or equibiaxial. Other approaches for forcing include compressive hydrostatic pressures applied to cells in culture, centrifugal forces applied by rotating cells and shear forces applied by fluid flow over adherent cells [25].

As proliferation of PDLFs helps regenerate periodontal tissue, several studies have examined the effects of strain on PDLF proliferation. Early reports suggested that cyclic strain increases the proliferation of PDLFs [26]. The effect of cyclic strain on cell proliferation in PDLFs appears to depend on the level of strain. For example, low levels of strain (2.5%) increased DNA synthesis in PDLFs, suggesting that strain can support proliferation [26]. However, larger strain magnitudes inhibit PDLF proliferation [27]. Cyclic strain can also induce apoptosis [28,29], particularly at pathological levels of strain (approx. 20% cyclic strain) [30]. Expectedly, the effects of strain on PDLF proliferation involve the mitogen-activated protein kinase (MAP kinase) pathway [31], but may also involve the Yes-associated protein (YAP) signalling pathway in a MAP-kinase-independent manner [32,33]. By contrast, centrifugal force has been reported to have no effect on PDLF proliferation [34]. Again, the type and magnitude of mechanical loading seem to reveal different pathways of PDLF mechanotransduction.

PDLFs secrete collagen [35,36] but also possess osteoblastic features, such as high levels of alkaline phosphatase activity and osteonectin [37,38]. Compressive stresses inhibit PDL cell proliferation [39,40]. Tensile strain applied to gingival fibroblasts promotes their proliferation [41], probably by increasing extracellular signal-regulated kinase phosphorylation, and these effects are abolished by inhibiting myosin activity [42].

## 3. The role of the cytoskeleton and cell–matrix adhesions in periodontal cell response to mechanical stress

Under uniaxial cyclic mechanical strain, PDLFs display the classic response of fibroblasts and endothelial cells to such strain: they reorient such that their long axis is perpendicular to the strain magnitude. Cell reorientation minimizes the strain put on the cell itself, and involves the remodelling of the F-actin cytoskeleton [43] and cell–substrate adhesions. Cells transmit tension generated in the actomyosin cytoskeleton to the substrate through sites of adhesion with the underlying substratum [44,45]. The small guanosine triphosphatase (GTPase) Rho controls F-actin assembly [46], and through its effector Rho-associated kinase and/or mDia1 [44], controls the phosphorylation of myosin light chain [47]. Rho thus regulates the level of intracellular tension, and also the assembly of cell–substrate adhesions [48]. The magnitude of intracellular tension and the extent of adhesion between the cell and the substratum determines the extent to which tensile strain applied to the underlying flexible substratum impacts intracellular signalling and response. For example, the extent to which endothelial cells reorient under uniaxial strain depends on the level of intracellular tension, and how strongly cells adhere to the underlying substratum [49,50].

Mechanical strain applied to the substratum activates RhoA in PDLFs [51], similar to observations in other cell types such as capillary endothelial cells [49] and vascular smooth muscle cells [52]. Conversely, compression decreases RhoA signalling, which may drive odontogenesis during embryonic tooth formation [53]. Compressive stresses cause RhoE GTPase-dependent disassembly of actomyosin stress fibres in PDL cells [54]. Centrifugal force activates Rho together with focal adhesion kinase in PDLFs [55]. How these different types of mechanical stresses differentially affect Rho needs further exploration.

A primary function of fibroblasts in the periodontium is secreting matrix proteins such as type 1 collagen, and organizing secreted matrix proteins into tensed and aligned networks. Mechanical forces modulate the expression of a remarkable array of proteins from periodontal cells [56–58]. In particular, collagen synthesis increases under cyclic strain [56,58–60], increases under shear flow [61], decreases under compression [62,63] and increases under centrifugal force [64]. There is further complexity in the fact that strain modifies collagen synthesis differentially depending on the type of collagen (fibril forming versus not) [65].

Mechanical forces alter expression of periodontal matrix metalloproteinases (MMPs) which enable matrix remodelling. These include MMP-2 [58,66], MMP-1 and -14 [62,67] and MMP-13 [68]. Compression and tension can cause opposing responses from cells. PDLFs subjected to 10% cyclic equibiaxial tensional forces *in vitro* secrete increased levels of type I collagen and fibronectin [69], and increased MMP-2 as well as tissue inhibitor of matrix metalloproteinase-2 (TIMP-2). By contrast, compressive stress decreases type I collagen, increases MMP-2 expression and TIMP-2 is unchanged [69]. Centrifugal force increases type I collagen in gingival fibroblasts [64]. The mechanisms for differential responses of cells to tension versus compression remain unclear, but highlight the importance of considering the type of mechanical loading and cell type in understanding periodontal mechanotransduction.

## 4. Effects of mechanical loading on fibroblast-mediated remodelling of alveolar bone

PDLFs and gingival fibroblasts both have signalling functions in the periodontium. They secrete proteins that can attract osteoclast precursors, which fuse with the bone surface and promote bone resorption, and they can secrete proteins that inhibit osteoclastogenesis [70]. Both PDLFs and gingival fibroblasts secrete osteoprotegerin (OPG), which is an osteoclastogenesis-inhibitory molecule, and also express RANKL which stimulates osteoclastogenesis. PDLFs, in particular, have a key role in controlling alveolar bone remodelling [2].

Human gingival fibroblasts under centrifugal force express higher levels of OPG relative to RANKL, which inhibits osteoclastogenesis [71], and addition of the inflammatory cytokine interleukin (IL)-1β promotes this effect. In addition, centrifugal forces upregulate type I collagen and osteopontin expression by gingival fibroblasts [64]. Centrifugal forces also inhibit osteoclastogenesis [71] by PDLFs through upregulation of OPG.

Compressive stresses applied to PDLs tend to increase the expression of osteoclastic molecules. Compressive stresses cause increased expression of inflammatory molecules [72] and RANKL in PDLs [73–76]. Mechanical stresses can also affect the secretion of bone remodelling proteins other than RANKL and OPG [40]. For example, compressive stresses induce the expression of osteopontin in PDL cells (which requires the Rho-kinase pathway) [77], and this may promote bone resorption by activating osteoclasts. The expression of sclerostin increases (mediated by TGF-β) when intermittent compression is applied to PDLFs [78]. Compressive stresses applied to PDLFs upregulate ephrin-A2 which inhibits osteoblast differentiation [79].

Tensile strain has the opposite effect to compression; it promotes signals from PDLFs that contribute to osteogenesis. Tensile strain upregulates ephrin-B2 in PDLFs which can have a stimulatory effect on osteoblasts [80], the secretion of soluble osteoprotegerin [81] and TGF-β1, which stimulates osteoblast proliferation and thereby promotes bone formation [82]. However, there is at least one report that tensile strain can stimulate the production of the osteoclastogenesis stimulatory molecule IL-1β by PDL cells [83].

PDL cells can directly contribute to bone formation by differentiating into osteoblast-like cells. Mechanical strain applied to PDLs promotes this osteogenic differentiation [31,84–86], through an increase in the levels of c-fos and c-jun. Mechanical strain induce differentiations and mineralization of PDLs through the activation of glutamate signalling pathways [87]. Tensile stress due to orthodontic tooth movement *in vivo* increases osteoblastic differentiation [87]. Osteogenesis in PDL cells has been observed under fluid shear [88] and under centrifugal force [89].

Overall, compressive stresses tend to promote bone resorption, while other types of stresses (tensile strain and fluid shear) tend to promote bone formation. Why these two different types of forces elicit such differential responses remains unclear.

## 5. Effect of mechanical forces on periodontal stem cell differentiation

PDL stem cells (PDLSCs) reside in the periodontal vasculature and have similarities to bone marrow-derived mesenchymal stem cells [90]. PDLSCs function in the periodontium to regenerate tissues including the PDL and cementum [91]. Mechanical strain promotes PDLSC proliferation [92–94]. Interestingly, stem cells from healthy periodontium are more tolerant to mechanical strain than cells from patients with periodontitis [92], suggesting that mechanical stress on gums due to chewing can be detrimental once there is onset of periodontitis. Mechanical vibration (which is much different from cyclic strain) promotes the proliferation and osteogenic differentiation of PDLSCs [94]. PDLSCs can also differentiate into keratocytes when exposed to mechanical strain [95].

## 6. Matrix rigidity sensing in periodontium

Cells in most tissues in the body are sensitive to the ‘passive’ mechanical properties of the matrix, such as elasticity [96], in addition to external mechanical force. Fibroblasts typically respond to matrix rigidity through Rho-mediated alterations [97] in actin polymerization, intracellular tension and adhesion assembly [98]. Changes in Rho and cytoskeletal assembly mediate phenotypic sensitivity of cells to matrix rigidity. For example, cells spread less on soft substrates compared to stiff substrates. Softer substrates and very stiff substrates support low speeds of cell migration, and intermediate stiffnesses support maximal speeds. Matrix stiffness impacts a wide range of genes in fibroblasts [99].

The mechanical properties of the gingiva are variable depending on their spatial location [100]. Periodontal cells are sensitive to the elasticity of the adhesive substratum through effects on the degree of cell flattening, and secrete more extracellular matrix proteins such as collagen and fibronectin on substrates of higher rigidity [24]. Matrix rigidity sensing by cells has been hypothesized to be a contributory factor in scarless wound healing by gingival fibroblasts [101], and to also promote bone remodelling due to the local release of ATP [102] during PD progression [103].

The shape of the nucleus is coupled with that of the cell. Round cell shapes on soft substrates result in less flattened nuclear shapes, while on stiff substrates the nucleus tends to be elongated and flat [104]. Such shapes probably reflect differences in the mechanical force exerted on the nuclear surface [105], and altered nuclear forces in fibroblasts cultured on soft substrates may impact the expression of a wide variety of genes in fibroblasts [99]. It is significant that PDLFs in the progression of PD progressively acquire rounder nuclear shapes, due to a loss of elongated PDLF morphologies [103]. Further elucidation of how PDLFs respond to changes in the mechanical properties of the local micro-environment will require a reliable quantification of the local mechanical properties of the PDL, itself which is a challenging task [106].

## 7. Cell mechanics in periodontal disease

The mechanical properties of cells may play an important role in the progression and establishment of PD. Yaffe and co-workers proposed the strain relaxation hypothesis for explaining alveolar bone resorption in PD nearly 15 years ago [103], and have continued to argue for it in a series of more recent papers [107,108]. They proposed that relaxation of tension in collagen bundles due to collagen degradation in the gingiva stimulates alveolar bone resorption [103]. Such relaxation of tension occurs, for example, during surgical sectioning of marginal gingiva where collagen fibres are severed; alveolar bone resorption is observed after such surgical sectioning [109–112]. Discarding the severed gingiva prevents alveolar bone resorption [111], suggesting that cells in the relaxed gingiva are likely to signal and promote alveolar bone resorption. Since then, Binderman *et al.* [110] have shown that surgical severing of dento-gingival fibres and PDL alters the expression of a variety of genes in cells including the P2X4 receptor and extracellular ATP, which participate in alveolar bone loss. That strain relaxation on fibroblasts may promote bone resorption is also supported by observations that compression during orthodontic tooth movement causes local alveolar bone resorption in patients [113,114]. Also, human gingival fibroblasts cultured on collagen gels reduce in length by nearly 45% when the collagen gel is detached from the underlying dish, and upregulate RANKL and P2X7 expression [115]. That strain relaxation may be produced in PDLFs is supported by the observation that PDLFs round up after mucoperiosteal flap surgery [103], and PDLFs are also seen to become less elongated in PD models [116].

Overall, such *in vivo* and *in vitro* results appear to support the concept that changes in fibroblast cell shape might control fibroblast-mediated resorption of alveolar bone. Cell shape has been shown to regulate cell fate [117], cell function [118–121], development [122,123], angiogenesis and cancer progression [49,124]. A potential relationship between cell shape and osteoclastogenesis is therefore of fundamental interest to the field of mechanobiology, and it is possible that PD is a disease primarily of abnormal cell mechanotransduction [125].

Unlike healthy human PDLFs, PDLFs from human patients with periodontitis display pathologic response to compressive stress by expressing inflammatory cytokines such as IL-6 and different, MMPs, MMPs-1, -7, -9 and -16 [126]. Similar to the effects of compressive stresses on osteoclastic molecular secretion such as RANKL discussed above, incubation of PDLFs with bacteria also upregulates osteoclastic molecules such as RANKL [127]. Bacterial infection alters F-actin organization and cell–matrix adhesions in gingival fibroblasts [128–131]. It is likely that the altered actomyosin cytoskeleton is a significant component of the molecular mechanisms by which PD progresses. The convergence between molecular responses to bacterial and mechanical stimuli suggests the potential for synergy between them in PD. Future research efforts that focus on the interaction between bacterial and mechanical stimuli will improve understanding of PD, and help develop effective and targeted PD therapies.

## 8. Conclusion

The periodontium is a key human tissue in which cells are constantly exposed to mechanical stresses. Periodontal cell mechanotransduction is critically important for the maintenance and remodelling of alveolar bone and becomes abnormal in PD. Mechanical stress elicits different molecular responses depending on the nature (compression, tension, shear, etc.) and the magnitude of stress. Bacterial infection-mediated PD tends to elicit molecular responses in PDLFS that are similar to compressive stress. Prevention of PD and development of therapeutics against PD will benefit from studies of the interaction between bacterial pathogenesis and mechanobiology.

## Data accessibility

This article has no additional data.
